# Instructional videos for parents/guardians of children with lip and palate clefts: integrative literature review

**DOI:** 10.1016/j.jped.2024.02.004

**Published:** 2024-04-25

**Authors:** Débora Letícia Moreira Mendes, Lucas Gabriel Nunes Andrade, Davide Carlos Joaquim, Francisco Cezanildo Silva Benedito, Ana Caroline Rocha de Melo Leite, Virgínia Cláudia Carneiro Girão-Carmona

**Affiliations:** a Universidade Federal do Ceará, Faculdade de Medicina, Departamento de Morfologia, Fortaleza, CE, Brazil; b Universidade da Integração Internacional da Lusofonia Afro-Brasileira, Instituto de Ciências da Saúde, Centro-Redenção, CE, Brazil

**Keywords:** Health education, Videos, Parents or guardians, Lip and palate clefts

## Abstract

**Objective:**

This study aimed to review literature from the past five years, focusing on the use of educational videos as a guidance tool for parents and guardians of children with lip and palate clefts.

**Source of data:**

Conducted between April and July 2022, this integrative literature review was framed around the question: 'What is the evidence regarding the use of videos in health education for parents/guardians of children with cleft lip and palate? PICO strategy was used to develop the research. A literature search was undertaken across PubMed, Web of Science, Scopus, and the Virtual Health Library databases. Of the eight articles included in this review, four were sourced from the PubMed database, with three published in 2021.

**Summary of the findings:**

The findings indicated that YouTube videos were moderately satisfactory and met the needs of parents or legal guardians to a partial extent. The majority of the videos analyzed in this review were characterized by a moderate level of informational content. One study particularly underscored that the content of these videos only partially satisfies the requirements of the parents or legal guardians of the children.

**Conclusions:**

Nevertheless, such videos are considered viable alternatives for health education, offering numerous benefits yet facing challenges, primarily due to the scarcity of information on orofacial malformations.

## Introduction

Cleft lip and palate represent some of the most prevalent craniofacial anomalies. While the exact etiology of these malformations remains elusive, emerging evidence suggests a multifactorial cause involving both genetic predispositions and environmental influences, as outlined by Costa et al.^1^ These congenital anomalies occur globally at a significant rate, affecting approximately one in every 700 live births, as reported in recent specialized literature.^2^^,^^3^ In the Brazilian context, the incidence of cleft lip and palate ranges from 0.19 to 1.54 per thousand live births, indicating a notable variation within the national demographic.^4^

It is noteworthy that cleft lip and palate anomalies extend beyond mere esthetic implications, profoundly impacting functional and psychosocial dimensions. These anomalies commonly disrupt essential functions such as swallowing, mastication, auditory acuity, respiration, and dental arch integrity, often leading to a nasalized voice. Additionally, patients with these conditions are predisposed to a heightened risk of recurrent otitis media in early life, potentially resulting in hearing impairments and necessitating frequent hospitalizations. This multifaceted impact underscores the complexity and far-reaching consequences of cleft lip and palate anomalies.^5^

Rehabilitation for individuals with cleft lip and palate typically commences with surgical interventions. These surgeries are generally scheduled at around three months of age for lip clefts and at 12 months for palate clefts. However, as the child develops, additional surgical procedures may be required to further enhance both esthetic and functional outcomes. These subsequent interventions not only contribute to the physical rehabilitation of the child but also have a significant positive impact on the psychological well-being of both the child and their family.^6^

In light of these considerations, the aspiration of parenthood^7^ is often entwined with the idealization of a 'perfect' child. The reality of giving birth to a child with a malformation can starkly contrast with these expectations, potentially precipitating a profound sense of disappointment. This experience may initiate a psychological process akin to mourning, as parents grieve the loss of the anticipated, idealized child.^8^ Such emotional responses underscore the complex psychosocial challenges encountered by families navigating the reality of congenital anomalies.

Frequently, the care of newborns is fraught with uncertainties and doubts, a situation that can be particularly pronounced in families of children with congenital anomalies. Such circumstances often give rise to heightened levels of fear and anxiety as parents confront the myriad of new responsibilities associated with their child's care. The complexities and demands of these responsibilities are often further amplified when specialized treatment is necessitated by the malformation, presenting an even more daunting challenge for the families involved.^9^

Parents of children with malformations often experience diverse psychological reactions such as anxiety, confusion, depression, shock, and frustration post-diagnosis.^10^ These emotional challenges are intensified by feelings of self-blame. Parents typically feel unprepared for the care responsibilities, especially in continuing treatments started in the hospital. This unpreparedness is largely due to doubts about their ability to manage their child's ongoing care needs.

Rafacho et al.^11^ identified that parents' most frequent inquiries pertain to surgical procedures for children with cleft anomalies. In addition to surgical concerns, the study highlighted several other key areas of parental interest. These include child development (25.7 %), general nutritional needs (14.3 %), timing of tooth eruption (14.3 %), etiology of the cleft condition (11.4 %), accessing financial resources for treatment (11.4 %), and the risk of subsequent children being born with a cleft (8.6 %).

Recent research has demonstrated the efficacy of multimedia materials in enhancing the learning process within the healthcare domain.^12^^,^^13^ Specifically, Costa et al.^14^ reported a notable improvement in knowledge acquisition following the use of multimedia materials. This was evidenced by an increase in the minimum number of correct responses on evaluative questionnaires completed by participants after viewing such materials, indicating a substantial enhancement in their understanding of the content.

In the realm of Health Education, the development of multimedia materials has emerged as a practice yielding positive outcomes. Research indicates that these materials are instrumental in enhancing skills that foster behavior conducive to health promotion and adherence to treatment protocols.^12^^,^^13^ This approach aligns with contemporary educational strategies, emphasizing interactive and engaging learning experiences to optimize health outcomes.

From this viewpoint, providing comprehensive guidance to caregivers of patients with cleft lip and palate, coupled with multidisciplinary follow-up, is pivotal for the holistic development of these children. Such approaches not only facilitate more effective care but also enhance treatment adherence. Consequently, in light of the challenges faced by parents and children affected by oral clefts, this study undertook a review of contemporary literature. The objective was to review literature from the past five years, focusing on the use of educational videos as a guidance tool for parents and guardians of children with lip and palate clefts.

## Methodology

This study represents an integrative literature review, centered on the utilization of instructional videos for parents and guardians of children with cleft lip and palate. A key aspect of this research involved identifying existing gaps within the current body of literature. Moreover, it entails a critical analysis of the available scientific evidence pertaining to the subject under investigation, as outlined in recent studies.^15^

The methodology of this review comprised six distinct steps: firstly, identifying the central theme and formulating the research question; secondly, selecting appropriate databases and defining descriptors; thirdly, conducting database searches and organizing the results using Rayyan Software;^16^ fourthly, selecting studies based on predetermined inclusion criteria; fifthly, evaluating the chosen studies and extracting relevant data; and finally, synthesizing and interpreting the data. This study employed the PICO strategy to structure the research question, addressing the following elements: Population (P) – parents of children with cleft lip and palate; Intervention (I) – health educational videos; Comparison (C) – not applicable; and Outcome (O) – evidence. The research question thus formulated was: 'What is the evidence regarding the use of videos in health education for parents/guardians of children with cleft lip and palate?'

In April 2022, a comprehensive search was conducted across several prominent databases, namely PubMed, Web of Science, Scopus, and the Virtual Health Library (VHL). The selection of descriptors was based on the structured vocabulary found in the Health Sciences Descriptors in English, including terms such as 'Cleft lip,' 'Cleft palate,' 'Orofacial cleft,' 'Needs assessments,' 'Videos,' 'Parents,' and 'Health education.' To facilitate a more refined and thorough search, Boolean operators AND and OR were strategically employed to cross-reference these descriptors across the aforementioned databases.

The inclusion criteria for this review stipulated that articles must be fully accessible, published within the last five years (2018–2022), and available in Portuguese, English, or Spanish. Exclusions were made for reviews, theses, dissertations, and anecdotal experience reports. The process of exporting studies was facilitated using Rayyan Software.^16^ Any duplicated articles identified during the review process were excluded. This selection and screening process was independently conducted by two researchers to ensure thoroughness and objectivity.

Subsequent to the selection process, the studies included in this review were meticulously organized. This organization entailed categorizing each study by database source, author/year of publication, study type, country of origin, journal title, level of evidence, research objectives, and principal results. The assessment of the level of evidence for each study adhered to the classification system proposed by Zina and Moimaz,^17^ which is as follows: level 1 for randomized clinical trials or systematic reviews; level 2 for cohort studies; level 3 for case-control studies; level 4 for cross-sectional studies, case reports, and case series; and level 5 for expert opinions/authority statements, as well as laboratory and animal studies.

## Results

**Figure 1:**
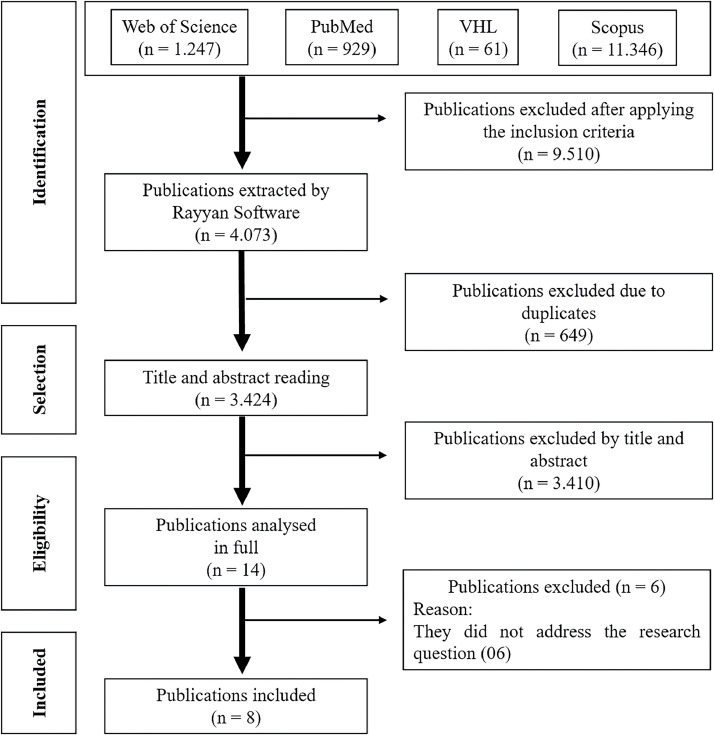
Identification of the study selection process to compose the integrative review. Fortaleza - CE, Brazil, 2022.

As data presented in Figure 1 indicate, the search strategy initially identified 13,583 publications. Following refinement for publication year and language, 9510 publications were excluded, leaving 4073. Subsequently, duplicate publications were removed, resulting in 3424 entries. After a thorough review of titles and abstracts, 3410 publications were further excluded. The remaining 14 publications underwent a comprehensive full-text assessment, leading to the exclusion of six publications that did not align with the research question. Ultimately, eight publications were integrated into the analytical framework of the review.

Within the scope of this review, eight studies were analyzed, of which four were identified in the PubMed database, with three of these published in 2021. The majority of the studies, totaling five, employed a descriptive research methodology. Geographically, both Türkiye and India contributed two articles each to this body of research. In terms of publication medium, three studies were featured in the Cleft Palate-Craniofacial Journal. Notably, five of these studies were categorized as level IV evidence, as detailed in Table 1.

**Table 1:** Characterization of studies included in the review, according to database, authorship and year, type of study, country, journal, and level of evidence. Fortaleza - CE, Brazil, 2022.

N°	Database	Author/Year	Type of Study	Country	Journal	L.E.*
1	PubMed	Bozkurt and Aras, 2021.	Descriptive retrospective study	Türkiye	Cleft Palate-Craniofacial Journal	IV
2	PubMed	Davies et al., 2018.	Descriptive study	United Kingdom	Pilot and feasibility studies	IV
3	Web of Science	Deshmukh et al., 2022.	Descriptive cross-sectional pilot study	India	Special Care In Dentistry	IV
4	Web of Science	Hakim et al., 2021.	Randomized clinical trial study	Iran	Global Pediatric Health	I
5	PubMed	Korkmaz and Buyuk, 2020.	Retrospective study	Türkiye	Cleft Palate-Craniofacial Journal	III
6	PubMed	Murthy et al.*,* 2020	Observational randomized clinical trial with concurrent parallel design	India	Special Care In Dentistry	I
7	Scopus	Razera et al., 2019.	Descriptive study	Brazil	Texto e Contexto Enfermagem	IV
8	VHL	Spoyalo et al., 2021	Qualitative descriptive study	Canada	Cleft Palate- Craniofacial Journal	IV

⁎ Level of Evidence.

Concerning the objectives of the studies included in this review, a notable diversity was observed. Three of the studies were primarily focused on describing the content and design of educational videos. In contrast, two other studies embarked on evaluating the influence and effectiveness of these videos in their respective contexts (Table 2).

**Table 2:** Presentation of titles and objectives of studies included in the review. Fortaleza - CE, Brazil, 2022.

N°	Title	Objective
1	Cleft lip and palate YouTube videos: content usefulness and sentiment analysis.	To describe the content of YouTube videos about lip and palate clefts and analyze the sentiment of related comments.
2	Development of an implementation intention-based intervention to change children's and parent-carers' behavior.	To describe a video animation design to teach parents and children to improve oral health.
3	Virtual reality as a parent education tool in pre‐surgical management of cleft lip and palate affected infants — A pilot study.	To evaluate the influence of virtual reality on parents' acceptance of nasoalveolar molding as pre surgical care for infants affected by lip and palate clefts.
4	The effect of combined education on the knowledge and care and supportive performance of parents with children with cleft lip and palate: A clinical trial study.	To investigate the effect of combined education on knowledge, care, and support performance in parents of children with lip and palate clefts.
5	YouTube as a patient-information source for cleft lip and palate.	To evaluate the content and quality of popular YouTube videos about lip and palate clefts treatment.
6	Assisted breastfeeding technique to improve knowledge, attitude, and practices of mothers with cleft lip- and palate-affected infants: a randomized trial.	To compare the effectiveness of the audiovisual module designed over the traditional instructional module in improving assisted breastfeeding habits.
7	Construction of an educational video on postoperative care for cheiloplasty and palatoplasty.	To describe the development of an educational video about the postoperative care of primary cheiloplasty and palatoplasty surgeries.
8	Online cleft educational videos: parent preferences.	To determine what parents of children with lip and palate clefts value in online educational videos and assess whether their needs are currently being met.

Overall, the majority of the videos analyzed in this review were characterized by a moderate level of informational content, as gauged by viewer satisfaction. In terms of audiovisual quality, the majority were evaluated as average or above. The publications further indicated that a smaller proportion of these videos were rated as moderately helpful or very helpful. Additionally, one study particularly underscored that the content of these videos only partially satisfies the requirements of the parents or legal guardians of the children.

Research assessing the efficacy of these videos has demonstrated their effectiveness in health education concerning cleft lip and palate. Key outcomes included enhanced understanding of the condition, improved caregiving practices, and expedited adaptation to breastfeeding techniques. As for the development of these videos, the literature suggests the utility of an advisory group, incorporating informal consultations with affected children and their parents. Nonetheless, it is noteworthy that only one study reported on the systematic development and validation of educational video content by expert judges in this field (Table 3).

**Table 3:** Description of the main results of the studies included in the review. Fortaleza – CE, Brazil, 2022.

N°	Main results
1	About 50 % of the videos were from the United States, and a third had no known country of origin. A quarter of the videos were sent by universities and hospitals; 22 % by health information sites; 17 % were provided by people with lip and palate clefts; 16 % by parents, and 15 % by health care providers. 49.1 % of the videos were educational and 48.2 described patients' experiences. Based on the 20 domains used for benchmarking, 31.5 % and 35.7 % of videos were rated as useful and very helpful, respectively. Related comment feelings include surgical pain, psychological issues, public embarrassment, and difficulty with physical appearance.
2	The team developed and refined the content and visuals with guidance from an advisory group and informal discussions with children in the target age group and their respective parents. The video explains how to formulate "if-then" action plans, starting from a conversation between a boy and his mother using the method, with oral health examples to illustrate the guidelines.
3	Most parents knew about feeding and how to assist in the feeding practice of infants affected by lip and palate clefts. However, only 33 % of participants in the control group were able to understand the physician's explanation of presurgical nasoalveolar molding. In comparison, 100 % of participants in the intervention group were able to visualize its benefits.
4	Forty parents of children with lip and palate clefts participated in the study, randomly divided into two groups of 20. After the educational intervention, the average score of parental care and support knowledge significantly increased in the intervention group compared to the control. There was also a significant difference in the average parental support performance score between the two groups.
5	There were fifty videos. 32 % were sent by a clinic; 54 % were classified as moderate informational content; 60 % were aimed at patient information; 54 % were classified as reasonable according to the satisfaction of the information provided, and the majority had average audiovisual quality or above.
6	There was a significant improvement in the mothers' knowledge from baseline to six months, however, practices indicated that mothers in the audiovisual module group had a better understanding of the condition and earlier adaptation to breastfeeding practices.
7	The evaluated items - content validity index referring to easy-to-understand and comprehensible language, good use of audiovisual resources, adequate transmission and distribution of content, maintenance of audience attention, facilitation of the memorization of messages, promotion of impact to stimulate attitudes and communication of the proposed objectives - were approved and agreed by most of the judges participating in the study.
8	Parents want accessible, trustworthy, relatable, and positive videos. They preferred short videos covering relevant topics as their child grows. The YouTube videos currently available only partially address these needs, such as hearing, teething, and surgeries for older children.

## Discussion

This study catalyzes a significant discourse on the role of health education, impacting various stakeholders including the academic community, healthcare professionals, parents/guardians, and children affected by cleft lip and palate. The primary impetus for this research is to delve into the scientific audiovisual materials available for educating parents/guardians. It aims to ascertain whether these resources effectively aid in comprehending the child's condition and provide guidance on optimal care practices. This study intends to explore the potential of these materials to mitigate further complications and enhance quality of life, an aspect underscored in several publications, thereby underscoring the criticality of this review.

The temporal distribution of the publications in this review underscores the contemporary relevance of the topic, with the year 2021 being particularly prominent. This recent focus aligns with the ongoing pertinence of cleft lip and palate as one of the most common congenital malformations, affecting approximately 1 in 700 live births.^18^ Additionally, there is an increasing research interest in enhancing parental education. The objective is to facilitate behavioral changes and improve the oral health outcomes for affected children.^19^

The form of the study being mainly descriptive is due to the researchers’ need to know the existing content. This type of design is essential to understand reality and to formulate hypotheses for clinical studies of articles.^20^

The geographical distribution of the publications in this review, predominantly from Türkiye and India, may reflect the higher prevalence rates of cleft lip and palate in the Asian continent. These countries' leading contributions to the literature could be correlated with the region's elevated incidence of the condition, reported as approximately 1 in every 500 live births in Asia.^18^

In terms of publication venues, the 'Cleft Palate-Craniofacial Journal' is noteworthy as the first international, interdisciplinary, peer-reviewed journal dedicated to advancing research in the care and treatment of individuals with cleft lip and palate, as well as other craniofacial anomalies.^21^ The majority of the studies in this review were categorized as level IV evidence, which is considered lower on the hierarchy of evidence. This categorization reflects the nature of level IV evidence, often characterized by deductive reasoning, a propensity for absolute assertions, and a degree of uncertainty. Such attributes may be less desirable for decision-makers seeking assertive and unequivocal guidance.^17^

The objectives of the studies within this review varied, with a significant emphasis on describing the content^22^ and the development process of educational videos.^19^^,^^23^ Given the increasing preference for online videos as a primary source of information about cleft conditions, the assurance of quality in the online materials available for parent and guardian education was identified as a critical concern.^22^ Additionally, several studies focused on assessing the impact and effectiveness of these videos were also noted.^24^^,^^25^

The investigated studies express reservations about the caliber of internet-based video resources on specific medical topics. In a detailed sentiment analysis of 112 YouTube videos, Bozkurt and Aras^22^ discerned that only 31.5 % and 35.7 % of these videos were of moderate and high utility, respectively. Complementing this, Korkmaz and Buyuk^25^ conducted an evaluation of the informational content and overall quality of widely viewed YouTube videos concerning the treatment of lip and palate clefts. Their findings predominantly categorized these videos as either of moderate or substandard quality. Based on this collective evidence, it becomes apparent that YouTube, as it stands, falls short of being a dependable informational resource for parents or guardians of patients in treatment for lip and palate clefts. This underscores the necessity for direct consultation with medical specialists to obtain accurate and reliable guidance.^22^^,^^25^

In their comprehensive survey, Spoyalo et al.^26^ explored the preferences of parents of children with lip and palate clefts regarding online educational videos. The findings revealed a distinct inclination towards videos that are not only accessible and reliable but also relatable and imbued with a positive tone. This insight highlights the significance of video content in meeting the informational needs and preferences of this demographic. The study underscores the importance of tailoring online educational materials to align with the specific desires and requirements of its intended audience, suggesting that the nature and quality of video content play a critical role in its effectiveness as an educational tool.

The reviewed studies not only focus on the quality and content of videos but also highlight a preference among parents or guardians of children with lip and palate clefts for concise video formats.^26^ Souza et al.^27^ argue that shorter videos are not only associated with faster download speeds, ease of portability, and shareability but also enhance the likelihood of viewers consuming the content in its entirety.

Furthermore, the suitability of didactic materials to the educational level of the target audience is paramount, as it has a direct impact on the comprehension of the presented content.^28^ Deshmukh et al.^24^ found that only 33 % of the participants initially understood the doctor's explanation regarding presurgical nasoalveolar molding and its significance. However, following an intervention using virtual reality, all participants in this group were able to comprehend and visualize its benefits fully.

These insights underscore the vital role of health education in the context of parents of cleft patients. Effective educational strategies not only enhance understanding but may also significantly influence the acceptance of and adherence to the recommended treatment. This underlines the necessity of developing tailored educational resources that are both accessible and comprehensible to the intended audience.

Research focusing on the efficacy of video-based interventions indicates that health education through this medium can significantly enhance parental understanding, potentially leading to improved caregiving and expedited adaptation to breastfeeding practices. In a notable study, Murthy et al.^29^ evaluated the impact of an audiovisual module compared to traditional instructional methods on the enhancement of assisted breastfeeding habits. The findings from this comparison revealed that mothers exposed to the audiovisual module exhibited a more comprehensive understanding of the pertinent health condition. This evidence underscores the crucial role of audiovisual tools, particularly videos, in health education. It suggests that integrating these resources into educational strategies could be a highly effective approach to conveying complex medical information, thereby facilitating better health outcomes.

In their research, Hakim et al.^30^ conducted a study involving 40 parents of children with lip and palate clefts, demonstrating the efficacy of a combined educational approach, integrating lectures and videos. This method showed a significant increase in both the knowledge and practical skills of these parents. Post-intervention, the data revealed that the average score pertaining to parental care and supportive knowledge was notably higher in the group that received the combined education, as compared to the control group. Based on these findings, the authors advocate for the implementation of combined educational methods. They posit that such an approach is more effective in enhancing the understanding and practical capabilities of parents caring for children with lip and palate clefts, suggesting a paradigm shift in educational strategies within this context.

Razera et al.^23^ undertook the development and validation of educational videos focusing on the postoperative care of cheiloplasty and palatoplasty. These videos underwent a rigorous evaluation by a panel of judges and were subsequently deemed effective in facilitating the training of caregivers for postoperative scenarios. The study found that these educational videos were highly efficient in preparing parents and other caregivers for managing the post-surgical care of children undergoing these specific procedures. This research highlights the potential of targeted audiovisual educational tools in enhancing the competency of caregivers in specialized medical contexts, emphasizing the value of such resources in healthcare education and patient support systems.

Despite the extensive literature available, the scope of publications included in this review was constrained. This limitation can be attributed to the research being conducted across only four databases, which potentially led to the omission of some pertinent studies on the topic. Additionally, the specific combination of descriptors used in the search methodology may have influenced the breadth and depth of the findings. This highlights the importance of a comprehensive and strategically diversified search approach in literature reviews to ensure a wide-ranging and thorough exploration of the subject matter.

## Conclusion

This review conclusively finds that online videos, in their current state, do not provide a sufficiently reliable source for educating parents or guardians of children with lip and palate clefts. This underscores the critical need for consultation with medical specialists in these cases.

Furthermore, education facilitated through audiovisual resources has proven to be effective. It promotes greater adherence to proposed treatments, facilitates early adaptation to breastfeeding practices, enhances knowledge, and improves the quality of care provided by parents and guardians.

The production of videos specifically tailored to the needs of parents and other caregivers of children with orofacial malformations is vital. However, it is imperative that these videos undergo a rigorous validation process involving expert judges. This step is essential to ensure the accuracy, quality, and effectiveness of the information conveyed.

## Funding

This work was supported by the Fundação Cearense de Apoio ao Desenvolvimento Científico e Tecnológico – FUNCAP (MLC-0191-00248.01.00/22).

## Authors’ contributions

Débora Letícia Moreira Mendes: Conceptualization, data curation, formal analysis, investigation, methodology, validation, visualization, writing – original draft, writing – review & editing.

Lucas Gabriel Nunes Andrade: Validation, visualization, writing - original draft.

Davide Carlos Joaquim: Data curation, formal analysis, investigation, methodology, validation, visualization, writing – original draft, writing – review & editing.

Francisco Cezanildo Silva Benedito: Validation, visualization, writing - original draft.

Ana Caroline Rocha de Melo Leite: Project administration, supervision, validation, visualization, writing – review & editing.

Virgínia Cláudia Carneiro Girão-Carmona: Project administration, resources, supervision, validation, visualization, writing – review & editing.

All authors participated in validation, visualization, writing – review & editing.

## Conflicts of interest

The authors declare no conflicts of interest.

## References

[1] Costa V.C., Silva RC da, Oliveira IF de, Paz L.B., Pogue R., Gazzoni L (2018). Aspectos etiológicos e clínicos das fissuras labiopalatinas. Rev Med Saúde Brasília.

[2] Morzycki A., Nickel K., Newton D., Ng M.C., Guilfoyle R. (2022). In search of the optimal pain management strategy for children undergoing cleft lip and palate repair: a systematic review and meta-analysis. J Plast Reconstr Aesthet Surg.

[3] Fonseca-Souza G., Oliveira L.B., Wambier L.M., Scariot R., Feltrin-Souza J. (2022). Tooth abnormalities associated with non-syndromic cleft lip and palate: systematic review and meta-analysis. Clin Oral Investig.

[4] Ré AF da, Schilling G.R., Sepúlveda C de los A.V., Silva C.M., Ferreira C.P., Pacheco G. (2022). Programa de Extensão para atendimento das fissuras labiopalatinas: atendimento fonoaudiológico. Res Soc Dev.

[5] Sousa G.F., Roncalli A.G. (2021). Fatores associados ao atraso no tratamento cirúrgico primário de fissuras labiopalatinas no Brasil: uma análise multinível. Ciênc Saúde Coletiva.

[6] Troíjo M.A., Tavano L.D., Rodrigues O.M. (2006). Enfrentamento de pais e mães de pacientes portadores de fissura labiopalatal durante à espera da cirurgia. Pediatr Mod.

[7] Caetano L.C., Scochi C.G., Angelo M. (2005). Vivendo no método canguru a tríade mãe- filho-família. Rev Latino-Am Enferm.

[8] Nusbaum R., Grubs R.E., Losee J.E., Weidman C., Ford M.D., Marazita M.L. (2008). A qualitative description of receiving a diagnosis of clefting in the prenatal or postnatal period. J Genet Couns.

[9] Guiller C.A., Dupas G., Pettengill M.A. (2009). Suffering eases over time: the experience of families in the care of children with congenital anomalies. Rev Latino-Am Enferm.

[10] Nelson J., O'Leary C., Weinman J. (2009). Causal attributions in parents of babies with a cleft lip and/or palate and their association with psychological well-being. Cleft Palate Craniofac J.

[11] Rafacho M.B., Tavano L.D., Romagnolli M., Bachega M.I. (2012). Hotsite de psicologia: informações de interesse sobre anomalias craniofaciais. Estud Psicol.

[12] Alencar C.J. (2008).

[13] Spinardi A.C. (2009). Procedimentos Terapêuticos no Transtorno Fonológico.

[14] Costa TL da C, Souza OM de, Carneiro H.A., Netto C.C., Pegoraro-Krook M.I., Dutka J de C.R. (2016). Multimedia material about velopharynx and primary palatoplasty for orientation of caregivers of children with cleft lip and palate. Codas.

[15] Botelho L.L., Cunha CC de A., Macedo M. (2011).

[16] Ouzzani M. (2016). Rayyan — a web and mobile app for systematic reviews. Syst Rev.

[17] Zina L.G., Moimaz S.A. (2012). Odontologia baseada em evidência: etapas e métodos de uma revisão sistemática. Arq Odontol.

[18] Dixon M.J., Marazita M.L., Beaty T.H., Murray J.C. (2011). Cleft lip and palate: understanding genetic and environmental influences. Nat Rev Genet.

[19] Davies K., Armitage C.J., Lin Y.L., Munro J., Walsh T., Callery P. (2017). Development of an implementation intention-based intervention to change children's and parent-carers’ behaviour. Pilot Feasibility Stud.

[20] Oliveira GJ de, Oliveira ES de, Leles C.R (2007). Survey of study design of papers published in Brazilian dental journals. Rev Odonto Ciência.

[21] Sage Journals (2022). https://journals.sagepub.com/description/CPC.

[22] Bozkurt A.P., Aras I. (2021). Cleft lip and palate YouTube videos: content usefulness and sentiment analysis. Cleft Palate Craniofac J.

[23] Razera A.P., Trettene A.D., Mondini C.C., Cintra F.M., Razera F.P., Tabaquim M.D. (2019). Construction of an educational video on postoperative care for cheiloplasty and palatoplasty. Texto Contexto Enferm.

[24] Deshmukh S., Murthy P.S., Singh B. (2022). Contractor I. Virtual Reality as parent education tool in pre-surgical management of cleft lip and palate affected infants—a pilot study. Spec Care Dentist.

[25] Korkmaz Y.N., Buyuk S.K. (2020). YouTube as a patient-information source for cleft lip and palate. Cleft Palate Craniofac J.

[26] Spoyalo K., Courtemanche R.J., Henkelman E. (2021). Online cleft educational videos: parent preferences. Cleft Palate Craniofac J.

[27] Souza C.F., Ferreira J.M., Pereira A.C., Silva M.A. (2019). Entendendo o uso de vídeos como ferramenta complementar de Ensino. J Health Inform.

[28] Lima M.B., Rebouças C.B., Castro R.C., Cipriano M.A., Cardoso M.V., Almeida P.C. (2017). Construction and validation of educational video for the guidance of parents of children regarding clean intermittent catheterization. Rev Esc Enferm USP.

[29] Murthy P.S., Deshmukh S., Murthy S. (2020). Assisted breastfeeding technique to improve knowledge, attitude, and practices of mothers with cleft lip- and palate-affected infants: a randomized trial. Spec Care Dentist.

[30] Hakim A., Zakizadeh Z., Saki N., Haghighizadeh M.H. (2021). The effect of combined education on the knowledge and care and supportive performance of parents with children with cleft lip and palate: a clinical trial study. Glob Pediatr Health.

